# Natural language inference for Malayalam language using language agnostic sentence representation

**DOI:** 10.7717/peerj-cs.508

**Published:** 2021-05-04

**Authors:** Sara Renjit, Sumam Idicula

**Affiliations:** Department of Computer Science, CUSAT, University in Kochi, Kochi, India

**Keywords:** Word Embeddings, LASER, Doc2Vec, FastText, BERT, Natural language inference, Malayalam

## Abstract

Natural language inference (NLI) is an essential subtask in many natural language processing applications. It is a directional relationship from premise to hypothesis. A pair of texts is defined as entailed if a text infers its meaning from the other text. The NLI is also known as textual entailment recognition, and it recognizes entailed and contradictory sentences in various NLP systems like Question Answering, Summarization and Information retrieval systems. This paper describes the NLI problem attempted for a low resource Indian language Malayalam, the regional language of Kerala. More than 30 million people speak this language. The paper is about the Malayalam NLI dataset, named MaNLI dataset, and its application of NLI in Malayalam language using different models, namely Doc2Vec (paragraph vector), fastText, BERT (Bidirectional Encoder Representation from Transformers), and LASER (Language Agnostic Sentence Representation). Our work attempts NLI in two ways, as binary classification and as multiclass classification. For both the classifications, LASER outperformed the other techniques. For multiclass classification, NLI using LASER based sentence embedding technique outperformed the other techniques by a significant margin of 12% accuracy. There was also an accuracy improvement of 9% for LASER based NLI system for binary classification over the other techniques.

## Introduction

Natural language inference applies to any language processing applications, where semantics is involved in conveying the context information. The definition of NLI is in terms of a pair of expressions, namely Premise (p) and Hypothesis (h). The premise entails the hypothesis if the hypothesis infers its meaning from the premise. If the meaning inferred from the premise is just the opposite of the meaning conveyed in the hypothesis, then the premise-hypothesis pair is contradictory. If there is no inferred information present in the hypothesis, the premise-hypothesis pair remains neutral. The premise (p) and text (t) are terms used interchangeably. NLI was introduced as textual entailment and it is first defined by Dagan & Glickman (2004) as text (t) entails hypothesis (h) (h is a consequent of t), if the meaning of h, as interpreted in the context of t, can be inferred from the meaning of t.

The classical definition of textual entailment is: A text t entails hypothesis h if h is true in every circumstance of a possible world in which t is true (Chierchia & McConnell-Ginet, 2001). This definition seems very strict and is not useful for real-world applications. A more applied definition is: A text t entails hypothesis h if human reading h infers that h is most likely true. The definition is again quantified mathematically by Glickman (2006) using probabilities as hypothesis h is entailed by text t if

(1) P(histrue|t)>P(histrue)

An example of inference pairs in the English language is shown below in Table 1.

**Table 1:** Example NLI sentence pairs from SNLI dataset (Bowman et al., 2015).

Premise	Hypothesis	Label
A little girl picking up a watermelon from a pile.	A girl is buying a watermelon.	Neutral
A little girl picking up a watermelon from a pile.	A girl is picking up an orange.	Contradiction
A little girl picking up a watermelon from a pile.	A girl is picking an item up.	Entailment

Textual entailment recognition is now more commonly referred to as natural language inference. Recognition of entailment is one of the subfields in textual entailment. The other subfield is the generation of textual entailment, which deals with creating or generating entailed sentences from the premise sentence. Few works related to generation includes rule-based approaches (Nevĕřilová, 2014) and sequence to sequence models (Kolesnyk, Rocktäschel & Riedel, 2016).

Most of the works mainly focus on recognition, mainly due to its wide application in other NLP tasks. Recognition of textual entailment was considered a binary classification in the initial years with small-sized datasets. Rule-based methods and logic-based reasoning are the main approaches with small datasets. Later, with the growth in size and type of datasets, recognition is considered a three-way classification. Moreover, many machine learning methods apply to this problem.

As human interaction with computers in different languages has increased, we require natural language processing applications in almost all languages. Malayalam is one such language in which many people interact with the system nowadays and communicate with other people through online media like blogs, Facebook, and Twitter. Malayalam is a Dravidian language used in verbal and written form in the southern part of India, especially in Kerala. It is one of the Asian languages in which attempts to increase its resources is ever-growing. The highly agglutinated and inflectional properties of this language make its language processing a challenging task.

NLI is an integral part of many NLP applications. For example, in summarization, the summary has to be entailed by the main document. Also, we can identify redundant sentences that need to be avoided in summary using entailment recognition. In Information extraction, the extracted information has to be entailed by the source documents. It also has an application in Question Answering, where the supporting answers for the question should entail a student answer for a question. Machine translation is another application in which the translated sentence should semantically match the standard translation. Paraphrasing is also actually a bidirectional textual entailment process.

This work focused on classifying the given pairs of Malayalam sentences into contradiction, neutral, and entailed classes with different embedding techniques. Our contributions in this work include:

1. This is the first application of an NLI model to the Malayalam language.
2. Malayalam does not have a benchmark NLI dataset like Stanford Natural Language Inference (SNLI) dataset for English. Hence we have developed an NLI dataset for the Malayalam language in consultation with language experts at Malayalam University, Kerala.
3. LASER based sentence representation for Malayalam sets benchmark results for the language. Its performance is comparable with state of the art Cross-lingual Natural Language Inference (XNLI) dataset based results for other languages.

The remaining part of this paper has the following sections: Section “Related Work” discusses the previous related works. The next section, “Materials and Methods”, explains the datasets used and the classification methodologies. Section “Experiments” details the experimental setup and parameter settings for the task, and Section “Results” provides the outputs from the experimental setting, followed by analysis and inferences in Section “Discussion”. We conclude with final remarks and future directions in Section “Conclusion”.

## Related Work

Natural Language Inference started with the Recognition of Textual Entailment (RTE) challenge in 2005, with a small dataset. This challenge happened for many years, which has lead to growth in size and type of dataset. The main goal of RTE challenges was to promote a generic task that captures the semantic needs of almost every NLP applications (Dagan, Glickman & Magnini, 2005). Traditional methods employed hand-engineered features like stemming, POS tagging, named entity recognition, coreference resolution, polarity features, numeric value identification, and also depended upon external resources like WordNet, EuroWordNet, VerbNet, and FrameNet (Kapetanios, Tatar & Sacarea, 2013). Increasing the dataset has helped various machine learning techniques like Support Vector Machines, linear classifiers, and logistic regression for inference identification.

The various RTE challenges are RTE-1 in 2005, RTE-2 in 2006, RTE-3 in 2007, RTE-4 in 2008, RTE-5 in 2009, RTE-6 in 2010, RTE-7 in 2011. Recognizing Inference in Text (RITE) was organized by NII Testbeds and Community for Information access Research project (NTCIR-9) in 2011. In 2012 Cross-lingual Textual Entailment for Content Synchronisation (CLTE) was organized by SemEval-2012 and the same in 2013. In 2013, Joint Symposium on Semantic Processing focused on textual inference. In 2014, Symposium on Semantic Processing also took multilingual textual inference as its main topic. RepEval 2017, The Second Workshop on Evaluating Vector Space Representations for NLP deals with a shared task on inference classification using the MultiNLI dataset. The approaches used for natural language inference are of three types: Classical rule-based, machine-learning based, and deep-learning approaches (Bowman et al., 2015).

The different methods used for recognition of textual entailment are based on directional methods using lexical entailment conditions such as Text (T) entails Hypothesis(H) iff *P*(*H*|*T*)>*P*(*H*). Let $T=(t_{1},\ldots ,t_{n})$ and $H=(h_{1},\ldots ,h_{n})$, then

(2) $$P(H|T)=\prod_{i=1}^{m}P(h_{i}|T)$$

where *P*(*H*|*T*) is the entailment confidence (Glickman & Dagan, 2005). Other methods use transformations in dependency graphs of the premise to obtain dependency graphs of hypothesis with a minimum cost. Similarity based on bag of words and syntactic matching are few other methods for text entailment recognition (Gautam, 2014). Challenges from RTE-3 deals with three classes, namely entailment, contradiction, and unknown. Most RTE-based approaches used various tools to preprocess the data, such as tokenization, stemming, lemmatization and part-of-speech taggers, parsers, and named entity recognizers. Resources like WordNet, VerbNet, VerbOcean are also used in initial attempts of classification (Dagan et al., 2010).

Bowman et al. (2015) introduced the Stanford Natural Language Inference (SNLI) dataset, a large dataset of 570 K sentence pairs labeled as Entailment, Contradiction, and Neutral class. Edit distance and lexical classifier based algorithms evaluated this dataset. As there was considerable data, richer models using sentence embeddings based on the sum of the word representations, recurrent neural network, and long short term memory networks were also used to evaluate inferences.

SNLI has led to the application of deep learning techniques in this problem. The deep learning based techniques for RTE problem are of two types: sentence encoding based models and match encoding based models (Mishra & Bhattacharyya, 2018). Sentence encoding based models focus on sentence representations for matching text hypothesis pairs, while match encoding models match the sentence pairs without forming sentence representations. Learned vector representations from word and its various combinations and models such as recurrent neural networks (RNN), long short term memory (LSTM), and convolutional neural network (CNN) applies to sentence encoding based methods. Bidirectional LSTM (BiLSTM) with attention mechanism and average pooling are other works in sentence encoding models for natural language inference (Sun et al., 2017; Ghaeini et al., 2018).

In match encoding based models, all words in a sentence have equal weights, but the significant words in deciding the classes get more weights using the attention mechanism by Rocktäschel et al. (2015). BiLSTM based model for sentence representation using Inner Attention, giving more importance to context words, is proposed by Liu et al. (2016).

### Low resource languages

Data scarcity is the main challenge for NLI applications in low resource languages. In Hindi, the NLI dataset is created using recasted data from four text classification dataset sources and two step classification is done into entailed and not-entailed classes by Uppal et al. (2020). They have used Bag of Words, Sent2Vec, InferSent, and XLM-ROBERTa based sentence embeddings, which are then classified using an MLP classifier and achieved accuracy in the range of 73–74% for the four recasted datasets. Another dataset is created as code mixed NLI from Hindi-English code mixed conversations taken from Hindi movie reviews. It used mBERT based evaluation resulting in an accuracy of 57.82% (Khanujaa et al., 2020).

Lexical entailments in low resource languages like Japanese and Thai are attempted by detecting hypernymy in text pairs. Cross-lingual, multilingual and meta-learning paradigms are used here. Experiments were conducted for French, Chinese, Finnish, Italian, Thai, Japanese, and Greek (Yu et al., 2020). An NLI benchmark dataset and transformer based model was developed for Filipino language (Cruz et al., 2020).

Alignment based approaches are used in the Arabic language for textual entailment recognition (Boudaa, El Marouani & Enneya, 2019; Etaiwi & Awajan, 2020). RTE attempts with the Italian EVALITA dataset includes translation based approaches (Pakray et al., 2012).

### Different datasets

Many datasets are available in the English language, and few for Italian, Japanese, and other languages. However, no datasets are available for the Malayalam language for entailment recognition, which sets back its NLI related works. The following are some of the prominent datasets for NLI in other languages.

- **FraCaS** dataset is a test suite created by FraCaS Consortium in 1996, and it contained 346 English textual inference pairs for NLI (Cooper et al., 1996).
- **RTE datasets**, RTE-1, is a balanced dataset consisting of manually collected 1,367 English sentence pairs. The data was collected from different domains, like question answering, information retrieval, machine translation, and text summarization. RTE-2 dataset has 800 sentence pairs, RTE-3 has also 800 pairs, RTE-4 has 1,000 pairs, RTE-5 has 600 pairs, RTE-6 has 15,955, and RTE-7 has 21,420 sentence pairs respectively collected from similar domains mentioned above (Ghuge & Bhattacharya, 2014).
- **SNLI** (Stanford Natural Language Inference) dataset comprises 570 K English sentence pairs labeled as Entailment, Contradiction, and Neutral by human annotators using Amazon Mechanical Turk (Bowman et al., 2015).
- **MultiNLI** (Multi-Genre Natural Language Inference) dataset is a collection of 433 K sentence pairs with more coverage and difficulty than the SNLI dataset (Williams, Nangia & Bowman, 2017).
- **XNLI** (Cross-lingual Natural Language Inference) dataset is a crowdsourced collection from MNLI containing 7,500 English sentence pairs. The translations of these sentence pairs are available in 14 languages, namely French, Spanish, German, Greek, Bulgarian, Russian, Turkish, Arabic, Vietnamese, Thai, Chinese, Hindi, Swahili, and Urdu (Conneau et al., 2018).
- **ANLI** (Adversarial NLI) dataset is the very new benchmark dataset prepared by humans and computational models in the loop procedure (Nie et al., 2019).
- **SciTail**, Textual entailment dataset contains 27 K sentence pairs from the science question answering systems (Khot, Sabharwal & Clark, 2018).
- **SICK** (Sentence Involving Compositional Knowledge) dataset consists of 9.8 K English pairs, developed as part of a shared task in SemEval 2014 (Marelli et al., 2014).
- **EVALITA dataset** is the textual entailment dataset in the Italian language consisting of 800 sentence pairs used for the EVALITA-2009 shared task competition (Bos, Zanzotto & Pennacchiotti, 2009).
- **ArbTE** is an Arabic language textual entailment dataset consisting of 600 sentence pairs (Alabbas, 2011).
- **RITE datasets** (Recognizing Inference in TExt) datasets were developed as part of the NTCIR workshop for textual entailment in Japanese and Chinese languages (Pakray, Bandyopadhyay & Gelbukh, 2013).

## Materials and Methods

### Dataset

Since a benchmark dataset was not available for NLI in the Malayalam language, we have developed a new NLI dataset named MaNLI, in consultation with language experts of the Malayalam University, Kerala. Malayalam translations of English sentence pairs available in the SNLI dataset were used for this purpose. Three inferences, namely, Contradiction, Neutral, and Entailment, were considered.

**Figure 1:**
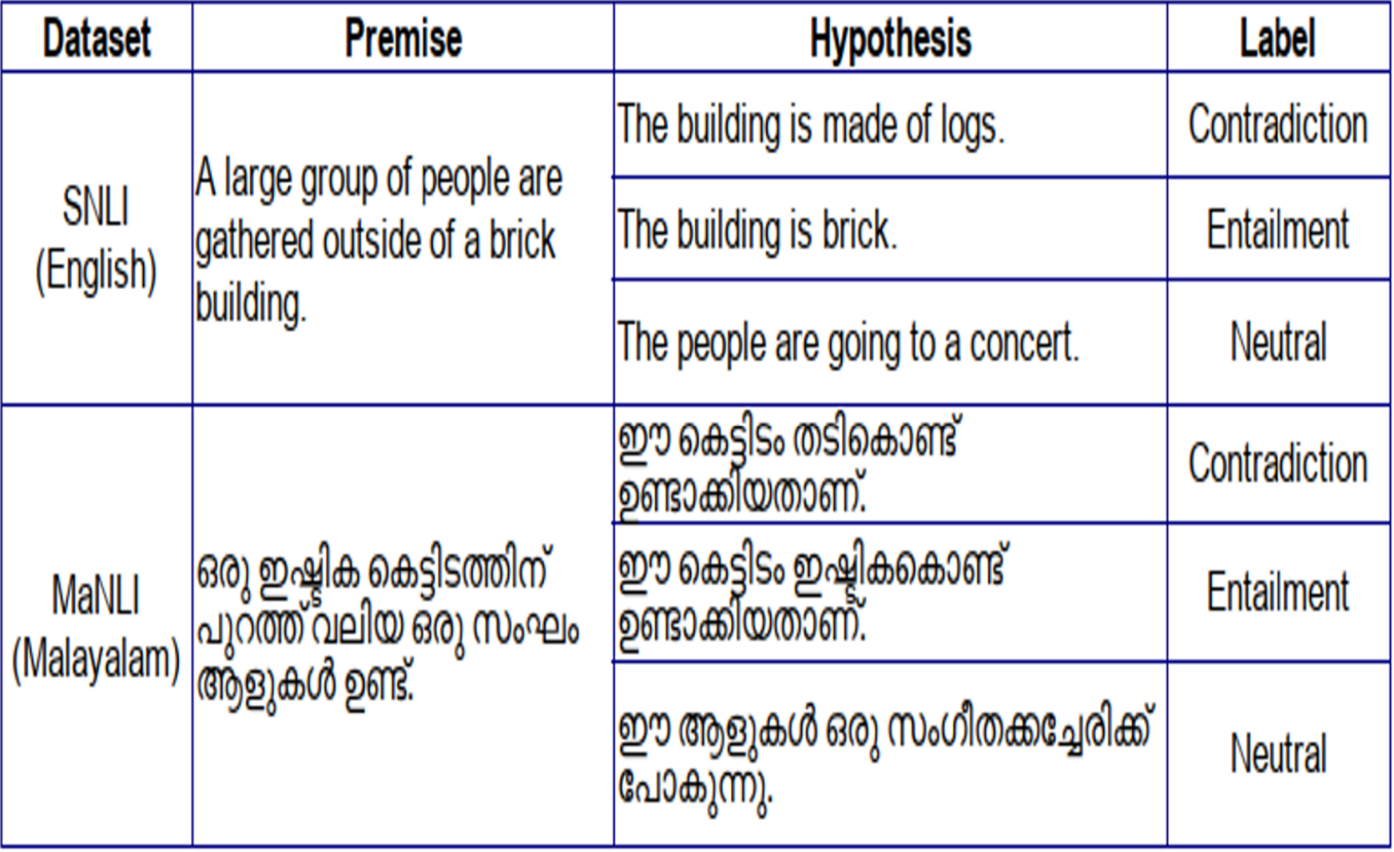
SNLI and MaNLI inference pairs.

This MaNLI dataset now contains 12 K Malayalam sentence pairs authenticated by language experts. Out of the 12 K sentence pairs, 4,026 are entailment pairs, 3,963 are contradiction pairs, and 4011 are neutral. Figure 1 shows an example of inference pairs in the MaNLI dataset and its reference in the SNLI dataset.

For the dataset preparation task, we have used automatic sentence translations using Google translator API, human translations and few language-specific word corrections using Olam dictionary (Nadh, 2013), which is an open-source English-Malayalam online dictionary. Samples from the SNLI dataset are translated into Malayalam language.

Linguistic changes such as word order corrections and few google translated malayalam words were replaced with appropriate words from Olam dictionary. This helped to maintain the same context information in the translated pairs with respect to English pairs. As we maintained the same context information, the same label annotations are used for the MaNLI pairs. A statistical summary of the MaNLI dataset in terms of unique tokens, average sentence length and lexical richness is provided in Table 2. A sample of 500 pairs from the MaNLI dataset was annotated by two manual annotators, and we obtained Cohen’s Kappa score (https://scikit-learn.org/stable/modules/generated/sklearn.metrics.cohen_kappa_score.html). value of 0.96, showing almost perfect agreement for the labels. We also used BLEU (Bilingual Evaluation Understudy: https://www.nltk.org/_modules/nltk/translate/bleu_score.html) based evaluation of the entailment pairs, contradiction pairs and neutral pairs to measure their ngrams overlap and the range of values obtained are depicted in Table 3.

**Table 2:** MaNLI dataset statistics.

	Premise	Hypothesis
Total no of tokens (*m*)	35,952	59,502
Total no of unique tokens (*n*)	9,172	12,234
Lexical richness (*n*/*m*)	0.2551	0.2056
Average sentence length	9.17	5.04

**Table 3:** MaNLI dataset evaluation using BLEU score.

Classes	Least score	Highest score
Entailment	0.007	1.0
Contradiction	0.002	0.96
Neutral	0.002	0.93

**Figure 2:**
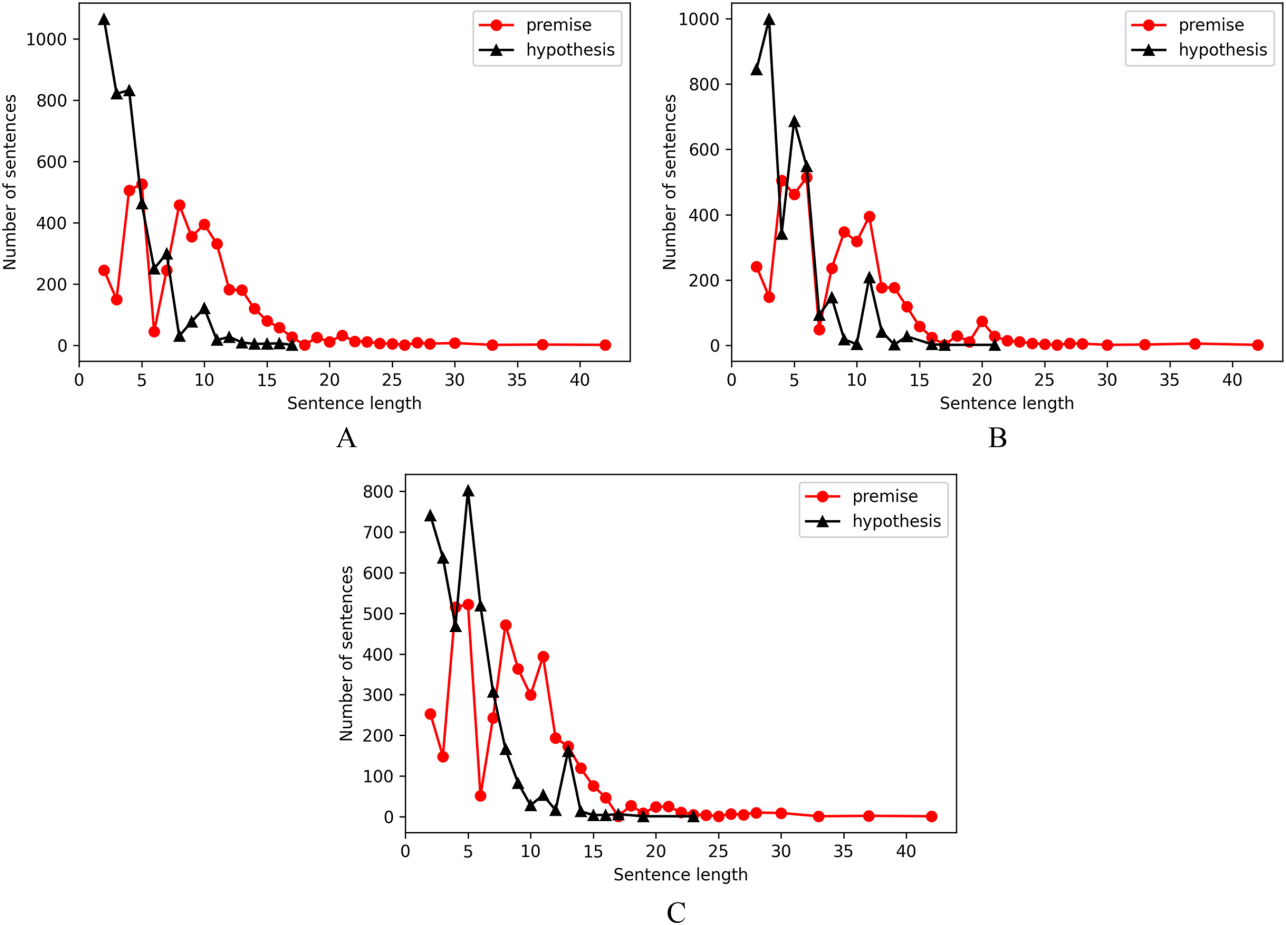
Sentence length distribution. (A) Entailment pairs. (B) Contradiction pairs. (C) Neutral pairs.

The sentence length of entailment pairs, contradiction pairs and neutral pairs are shown as separate distributions in Fig. 2, from which we observed that there are more number of instances of premise sentences with length lesser than or equal to 15 and hypothesis sentences with length less than or equal to 10 as compared with samples of length above 15 for premise and 10 for hypothesis.

## Methods

This section discusses the different sentence representations and the system architecture for inference classification in the Malayalam language. We dealt with the NLI problem in two ways, binary classification and three-way (multiclass) classification. Our method focused on how appropriate sentence representations helped in better classification. We used sentence representations using Doc2Vec (paragraph vector), fastText word vectors, BERT (Bidirectional Encoder Representations from Transformers), and LASER (Language Agnostic Sentence Representations).

### Sentence representation using Doc2Vec

Doc2Vec, also called paragraph vector (Le & Mikolov, 2014), is an unsupervised framework for learning distributed vector representations for variable-length sentences. This framework is derived from the Word2Vec model (Mikolov et al., 2013). Doc2Vec uses the same architecture as that of Word2Vec model (Rong, 2014) with an addition of randomly initialized sentence vector along with the context word vectors. The weight matrix between the sentence vectors in the input layer and the hidden layer is represented as a column matrix. The concatenated representation of sentence vector with vectors for words present in that particular sentence is fed to a classifier and is trained using stochastic gradient descent through backpropagation. Prediction of the most appropriate target word is done using a Softmax classifier. This method is the distributed memory model (PV-DM) in which the paragraph vector serves as a memory that remembers its context information and word order is also considered.

### Sentence representation using fastText

In this method, we used word vector representations from fastText (Bojanowski et al., 2017). It is available in many languages, including Malayalam. fastText provided pretrained word vector representations trained on Common Crawl and Wikipedia data in 294 languages, including Malayalam, which we utilized to create sentence representations. fastText model uses a continuous bag of words with character n-grams of length 5 to produce 300-dimensional representations. This model learns word representations by taking into account subword units, utilizing the morphological property. We use wiki word vectors as in the form of an embedding matrix to initialize our embedding layer, which produces word vectors for incoming words in each sentence. This sequence of word vectors is input to an LSTM layer and output sentence representations. The combined form of sentence pairs is then fed to a neural network with dense layers for softmax and sigmoid classification.

### Sentence representation using BERT

BERT (Bidirectional Encoder Representations from Transformers) (Devlin et al., 2019) has a transformer model (encoder-decoder architecture) pretrained on Wikipedia data from 104 languages including Malayalam. It is trained for two tasks: Masked language modeling and Next sentence prediction. We used the masked language modeling based pretrained model with 12 layers (transformer blocks), 768 as hidden size, 12 attention heads, and 110 M total parameters. 15% of words from a sentence are masked randomly, and the model is trained to predict these masked words. Thus this model learns bidirectional representations for sentences. Input is in the form of a sequence of tokens using WordPiece embeddings. The final input representation for a token is the sum of position embeddings, segment embeddings, and token embeddings.The sentence representation is obtained from the [CLS] token embedding.

### Sentence representation using LASER

**Figure 3:**
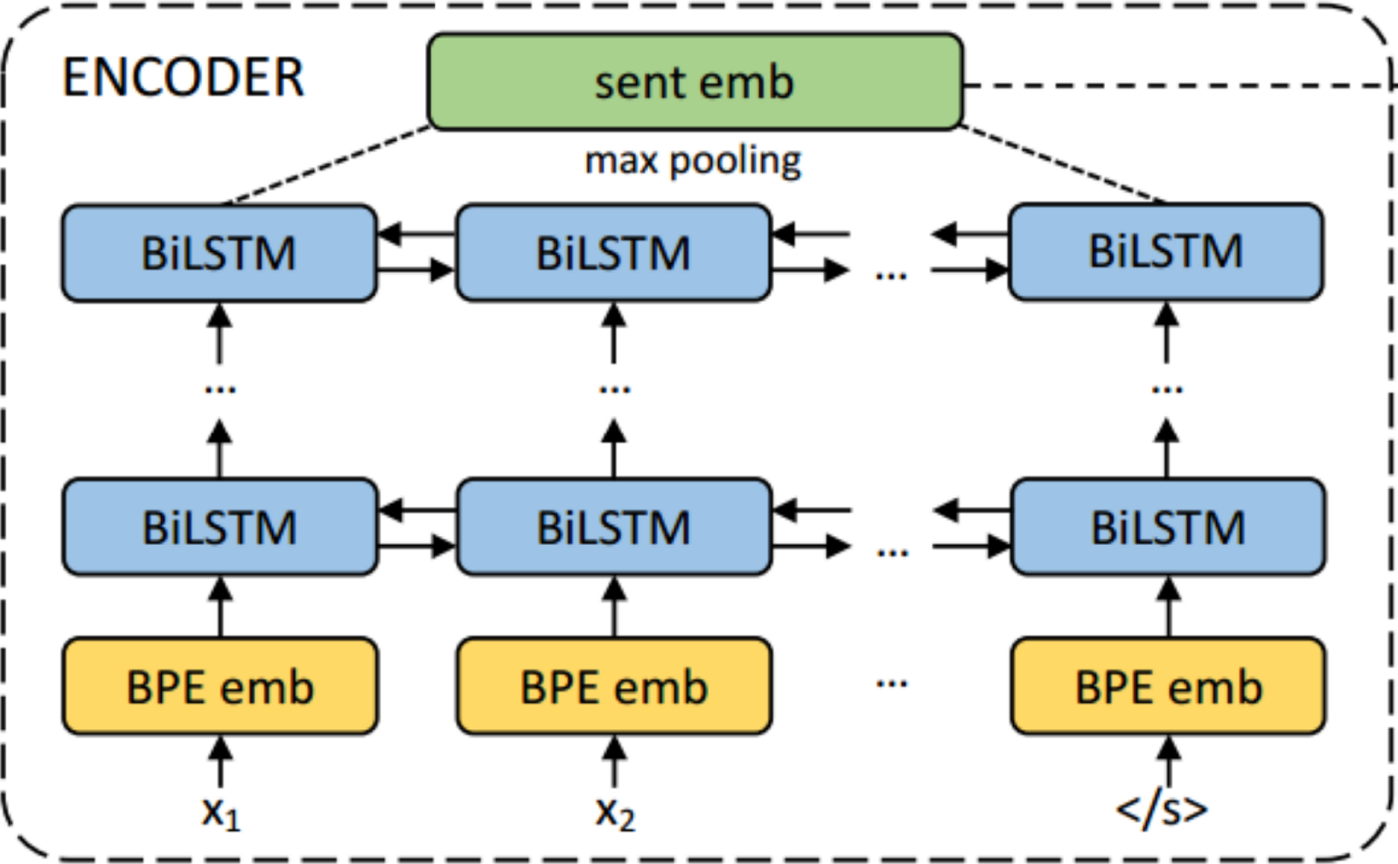
The encoder architecture of LASER encoder decoder system for multilingual sentence embedding (Artetxe & Schwenk, 2019).

LASER (Artetxe & Schwenk, 2019) based sentence encoder is a BiLSTM multilingual sentence encoder trained for 93 languages, including Malayalam. Figure 3 shows the architecture of the LASER system. The encoder-decoder is trained with parallel corpora where *x*_*i*_ represents the words from a sentence in one language and *y*_*i*_ are the words from the sentence translated in other languages. This system is trained as English → other languages and vice versa. This method uses a single encoder and decoder for all 93 languages based on a common vocabulary learned from the concatenation of all training data from the parallel corpus using joint byte pair encoding. This technique considers character and word level hybrid representations for large corpora. Semantically similar sentences irrespective of the language are mapped close in their embedding space using this approach.

The input sentence’s words are embedded using a joint byte pair encoding method, with a joint vocabulary shared for all languages. It is fed to a BiLSTM encoder with 1 to 5 layers of 512 dimensions, and a max-pooling operation is applied to obtain 1,024 dimensional sentence embeddings. No language information is used in the encoder during training, thereby making it a language independent encoder.

### System architecture

**Figure 4:**
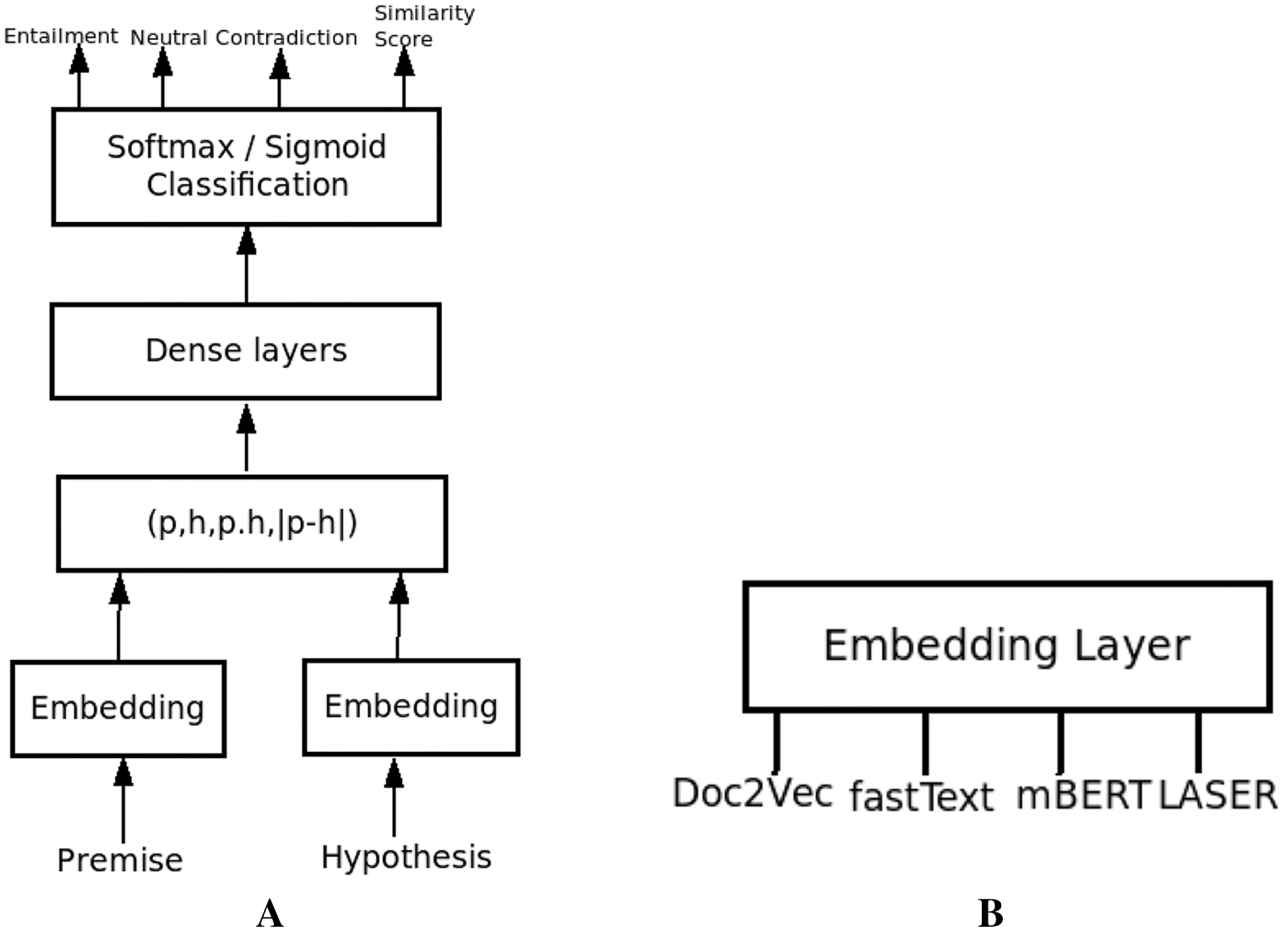
The NLI System. (A) NLI system architecture. (B) Different embedding approaches used.

We implemented an NLI system based on the sentence representations mentioned above using the general NLI system architecture, as shown in Fig. 4. The different modules in our system are the embedding module, where the raw premise and hypothesis sentences are represented in their embedded representations utilizing sentence embedding techniques using Doc2Vec, fastText, BERT and LASER. No preprocessing techniques are applied before embedding.

Sentence pair representation (*p*,*h*,*p.h*,|*p* − *h*|) is the concatenation of embedded premise and hypothesis representations, and their dot product and absolute difference forming the input representation to Dense Layer. Dense Layers consist of a feed-forward neural network with 1 to 2 dense layers with Relu activation, Adam optimizer, and cross-entropy loss function. The last layer is the output layer with three neurons and one neuron for multiclass and binary classification, respectively, using Softmax and Sigmoid activation functions. As part of this classification, for binary classification, we also obtain a probability value from sigmoid which can be used as a similarity score between premise and hypothesis. In case of multiclass classification, the probabilities from softmax classifier, help obtain a confidence score, which can be used to quantify the entailments, contradictions and neutral pairs. It also helps to identify the cohesiveness between sentences or semantic similarity, useful in other language processing applications like multidocument summarization and question answering systems.

## Experimental Results

The inference of Malayalam premise hypothesis pairs is done as a multiclass classification and a binary classification problem. Implementations used Google Colab CPU computing engine, Spyder environment with Python, Tensorflow and Keras frameworks for classification and Scikit-Learn Library for performance evaluations. The pretrained models of multilingual BERT and LASER are used to encode sentences in Colab environment, Doc2Vec and fastText based embeddings and all classifications are done in Spyder environment. Scikit-Learn based classification report and confusion matrix are used for error analysis.

In order to find the best sentence pair representation to feed as input to the classifier, we evaluated multiclass classifier with LASER based sentence embeddings for different sentence pair combinations. The representation in the form (*p*,*h*,*p.h*,|*p* − *h*|), where p and h denote premise and hypothesis, shows better performance as in Table 4 and hence, we fixed the same representation for all other classification. The form (*p*,*h*,*p.h*,|*p* − *h*|) is the concatenated representation of premise and hypothesis vectors along with their dot product and absolute difference. This sentence pair representation is used in other works also (Artetxe & Schwenk, 2019).

**Table 4:** Different sentence pair combinations.

Representation	Train accuracy	Test accuracy
(*p*,*h*)	0.609	0.546
(*p*,*h*,*p.h*)	0.600	0.548
(*p*,*h*,|*p* − *h*|)	0.689	0.630
(*p*,*h*,*p.h*,|*p* − *h*|)	0.721	0.642

For both types of classification, we implemented systems based on Doc2Vec, fastText word embeddings, BERT, and LASER sentence embeddings, and the experimental settings are given below.

- **Experimental setup for Multiclass classification**: MaNLI dataset is trained for classification into three classes, namely, Entailment, Contradiction, and Neutral. Out of 12 K premise hypothesis pairs, 7 K pairs were used for training and 5 K sentence pairs for testing. The embedded representation of sentences is trained using a neural network with fully connected layers. Relu activation is used in these dense layers, followed by softmax activation for classification, which classifies the output to the class, which has a maximum probability. Early stopping condition with patience values of 5, 10 are used to get the optimum performance.
- **Experimental setup for Binary classification**: This classifier used 7,989 pairs from MaNLI dataset, out of which 6,391 pairs are used for training and the rest for testing. The binary classification system also has the same network structure with sigmoid activation and binary cross-entropy loss. The training is stopped using the early stopping condition.
- **Sentence embedding using Doc2Vec**: The whole dataset consisting of sentence tags and their corresponding word collection is used to build a vocabulary from the set of sentences. The Doc2Vec is initialized with a minimum count as 1 to consider all words in the corpus, window_size 5, vector size 100 for output embedding dimension, and used the distributed memory approach. The model is trained for 10 epochs with random shuffle of the dataset for each iteration and initial learning rate of 0.025.
  Sentence vectors are inferred from this model for the MaNLI dataset. It is then passed to a neural network with size (400 × 200 × 3) in input, hidden and output layers. Relu activation is used for input and hidden layer and output layer classifies the data using Softmax for multiclass and Sigmoid for binary classes. The training is stopped in 3 epochs for multiclass and 4 epochs for binary classification. Table 5 shows the results of this model based on Doc2Vec embedding.
- **Sentence embedding using fastText word embeddings**: We used fastText (Bojanowski et al., 2017) wiki vectors in wiki.ml.vec to construct an embedding matrix, using which words in the sentences are embedded through the Keras Embedding layer and then encoded into sentence representation through an LSTM encoding layer. The individual premise and hypothesis representations are then combined and fed into a feed-forward neural network for classification. The embeddings from the LSTM layer for each sentence sequence is 300 dimensional in vector space. The sentence pair representation is fed to a feed-forward neural network with two dense layers of size (1,200 × 600). This neural network classifier’s output layer has (3,1) neurons for softmax and sigmoid classification, trained for 14 and 22 epochs, respectively. The NLI classifier results based on fastText based sentence representations are in Table 6.
- **Sentence Embedding using BERT**: For BERT based sentence embeddings, the tokenizer and encoder is initialized with the pretrained multilingual model, bert-base-multilingual-cased, which is already trained on 104 languages. The input sentences are tokenized through the BERT tokenizer module that embed each token with the sum of its position, segment and token embeddings. This is then passed to the encoder module and output corresponding to the [CLS] token is the embedded sentence representation. The sentence pair combination is then trained using a functional model neural network with layers sized (3,072 × 768 × 3) with Relu activation for input layer and hidden layer and the final layer has softmax for classification into entailment, contradiction and neutral classes. The training stops in 13 epochs with Adam optimizer and cross entropy loss function. For binary classification, the final layer has a sigmoid activation and the network is trained for 15 epochs. The classifier results using BERT based sentence embeddings are shown in Table 7.
- **Sentence embedding using LASER**: In this embedding technique, we used the bilstm.93langs.2018-12-26.pt pretrained model and each sentence (premise\hypothesis) is encoded in 1,024 dimensional vector representations. The combined representation of sentence pairs was used as input to train a sequential feed-forward neural network with dense layers sized (4,096 × 512 × 384 × 3) neurons. Relu activation is used in all layers except the final layer. The model is trained with Adam optimizer and cross-entropy loss. The training stops in 4 epochs for multiclass and 6 epochs for binary classification. The classifier results using LASER sentence representations are shown in Table 8.

**Table 5:** Results based on Doc2Vec for classification of MaNLI dataset.

	Multiclass_Doc2Vec				Binary_Doc2Vec			
	Precision	Recall	F1-score	Support	Precision	Recall	F1score	Support
Contradiction	0.43	0.67	0.53	1,651	0.56	0.77	0.65	798
Entailment	0.47	0.30	0.37	1,682	0.63	0.40	0.49	800
Neutral	0.64	0.53	0.58	1,667	–	–	–	–
micro avg	0.50	0.50	0.50	5,000	0.58	0.58	0.58	1,598
macro avg	0.51	0.50	0.49	5,000	0.59	0.58	0.57	1,598
weighted avg	0.52	0.50	0.49	5,000	0.59	0.58	0.57	1,598

**Table 6:** Results based on fastText for classification of MaNLI dataset.

	Multiclass_fastText				Binary_fastText			
	Precision	Recall	F1-score	Support	Precision	Recall	F1score	Support
Contradiction	0.58	0.40	0.47	1651	0.74	0.57	0.64	798
Entailment	0.50	0.72	0.59	1682	0.65	0.80	0.72	800
Neutral	0.52	0.45	0.48	1667	–	–	–	–
micro avg	0.52	0.52	0.52	5000	0.69	0.69	0.69	1598
macro avg	0.53	0.52	0.51	5000	0.70	0.69	0.68	1598
weighted avg	0.53	0.52	0.52	5000	0.70	0.69	0.68	1598

**Table 7:** Results based on BERT for classification of MaNLI dataset.

	Multiclass_BERT				Binary_BERT			
	Precision	Recall	F1-score	Support	Precision	Recall	F1score	Support
Contradiction	0.58	0.27	0.37	1,651	0.71	0.53	0.61	798
Entailment	0.45	0.77	0.57	1,682	0.63	0.79	0.70	800
Neutral	0.55	0.45	0.49	1,667	–	–	–	–
micro avg	0.53	0.50	0.48	5,000	0.67	0.66	0.65	1,598
macro avg	0.53	0.50	0.48	5,000	0.67	0.66	0.65	1,598
weighted avg	0.53	0.50	0.48	5,000	0.67	0.66	0.65	1,598

**Table 8:** Results based on LASER for classification of MaNLI dataset.

	Multiclass_LASER				Binary_LASER			
	Precision	Recall	F1-score	Support	Precision	Recall	F1score	Support
Contradiction	0.67	0.58	0.62	1,651	0.73	0.87	0.79	798
Entailment	0.62	0.79	0.70	1,682	0.84	0.68	0.75	800
Neutral	0.64	0.55	0.60	1,667	–	–	–	–
micro avg	0.64	0.64	0.64	5,000	0.77	0.77	0.77	1,598
macro avg	0.65	0.64	0.64	5,000	0.79	0.77	0.77	1,598
weighted avg	0.65	0.64	0.64	5,000	0.79	0.77	0.77	1,598

### Evaluation metrics used

The different metrics mainly used in this classification are based on the classification report using the Scikit-Learn library. An insight and better understanding of the classifier performance over each class rather than the global accuracy is obtained by using a classification report. Understanding the classifier behavior through recall and precision values helps put the system in different applications based on the systems’ precision-recall requirements. The metrics used are:

**Precision** Precision measures the correctness of the system, how precisely the model correctly classifies each class. It is defined as the ratio of correctly predicted instances for a class to the total number of predicted instances for that class, according to the equation,

(3) $$precision=\frac{TP}{TP+FP}$$

**Recall** Recall shows the ability of the classifier to find all positive instances. It is defined as the ratio of correctly predicted instances to the actual number of correct instances in each class. The recall is measured by,

(4) $$recall=\frac{TP}{TP+FN}$$

**F1-score** F1-score is defined as the weighted harmonic mean of recall and precision, and it is a measure to compare classifier models more appropriately. It is measured using the formula,

(5) $$F1-score=\frac{2*(Recall*Precision)}{\left(Recall+Precision\right)}$$

**Support** Support shows the number of actual instances of each class in the dataset.

**Accuracy** Accuracy is the ratio of correct inferences to the total inferences in the dataset. It is measured as,

(6) $$accuracy=\frac{TP+TN}{TP+FP+FN+TN}$$

TP is true positives, the number of positive instances correctly classified as positive. TN denotes true negatives, the number of negative instances correctly classified as negative. FP is false positive, the number of instances incorrectly classified as positive. FN is false negative, the number of instances incorrectly classified as negative.

**Figure 5:**
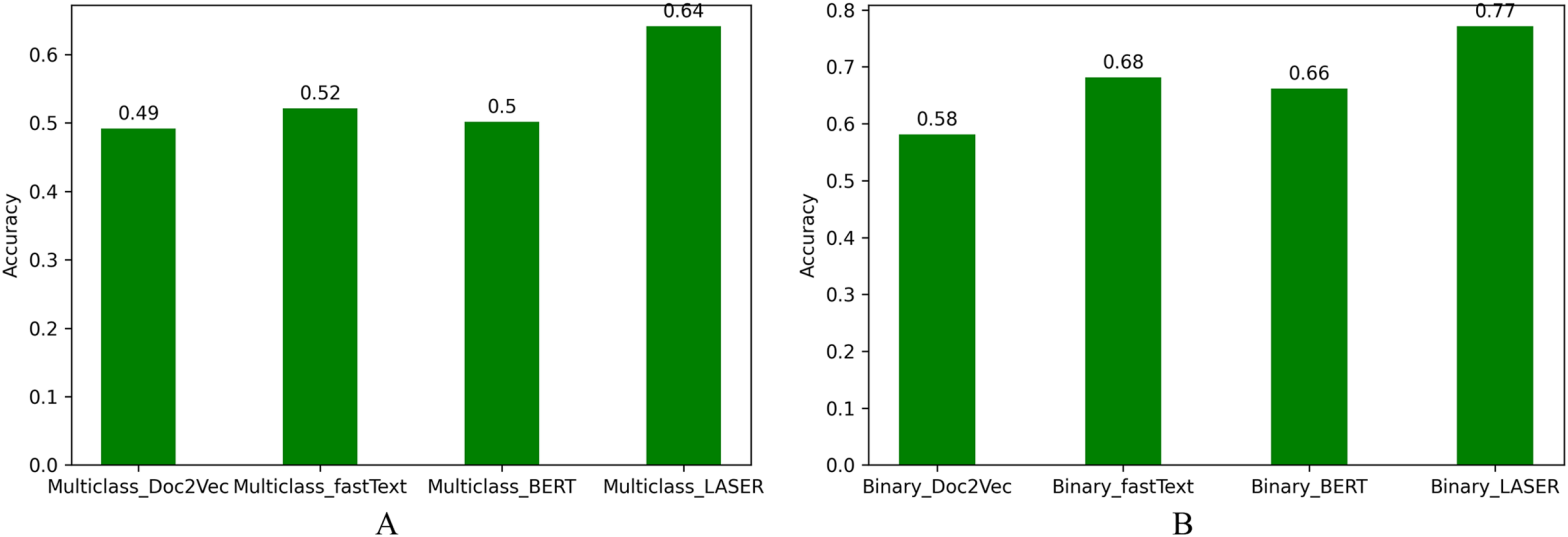
Accuracy for NLI classification. (A) Multiclass NLI system. (B) Binary NLI system.

Based on model predictability, the accuracy of different systems implemented in this work is compared in Fig. 5. We observe that accuracy of LASER based multiclass and binary classification systems is higher than the other embedding techniques.

### Baselines

Classifiers with word vector based sentence representations of 100 dimensions using Word2Vec model and LSTM encoder based Softmax classifier is used as baseline approaches for the SNLI dataset (Bowman et al., 2015). The MaNLI dataset is evaluated with the same approaches and the classification accuracy is shown in Table 9.

**Table 9:** Accuracy of baseline approaches compared with Doc2Vec, fastText, BERT and LASER.

Approach	Multiclass	Binary
100D Sum of words	0.36	0.56
100D Mean of words	0.42	0.63
100D LSTM	0.33	0.50
100D Doc2Vec	0.49	0.58
300D fastText	0.52	0.68
768D mBERT	0.50	0.66
1024D LASER	0.64	0.77

### Comparison with other languages

When the four methods are compared, LASER based method is better than other approaches for the MaNLI dataset. The English version of the test corpus is also tested using the LASER embedding based classification model. The LASER embedding based results for the English version of the dataset having a total of 12,000 sentence pairs are shown below in Table 10 in terms of its weighted average precision, recall and F1-score. It has an accuracy value of 0.67 for multiclass and 0.84 for binary classification. The results are similar to that for Malayalam language and hence we observe that model performance can be appreciated using the LASER based method.

**Table 10:** Results based on LASER for classification of English version of MaNLI dataset.

	Multiclass_LASER				Binary_LASER			
	Precision	Recall	F1-score	Support	Precision	Recall	F1score	Support
Contradiction	0.67	0.70	0.69	1,651	0.84	0.84	0.84	798
Entailment	0.65	0.83	0.73	1,682	0.84	0.84	0.84	800
Neutral	0.71	0.48	0.57	1,667	–	–	–	–
micro avg	0.68	0.67	0.67	5,000	0.84	0.84	0.84	1,598
macro avg	0.68	0.67	0.67	5,000	0.84	0.84	0.84	1,598
weighted avg	0.68	0.67	0.67	5,000	0.84	0.84	0.84	1,598

BERT based evaluation of datasets of English and other Indian languages is in Table 11 and we extended this with our multilingual BERT model for the MaNLI dataset. RTE, SNLI, MNLI, QNLI are English datasets, NLI En-Hi is English Hindi code mixed dataset and MaNLI is Malayalam NLI dataset. SNLI, MNLI and QNLI are English datasets that used BERT base pretrained model, which is already trained with large corpus in English language. RTE, NLI En-Hi and MaNLI are small sized as compared with SNLI and hence their performance drops.

**Table 11:** BERT based comparison of MaNLI with other datasets (Khanujaa et al., 2020).

Dataset	Model	Accuracy
RTE	BERTbase	66.4
SNLI	BERTbase	90.4
MNLI	BERTbase	86.7
QNLI	BERTbase	90.5
NLI En-Hi	mBERT	57.82
**MaNLI**	mBERT	50.0

Table 12 shows the test accuracies of BiLSTM (Conneau et al., 2018), BERT (Devlin et al., 2019), and LASER (Artetxe & Schwenk, 2019) tested on XNLI dataset. From Table 12, LASER based systems have accuracy values in the range of 61.0% to 73.9%. We can infer that our LASER based system is comparable with the XNLI results. Thus LASER embeddings capture more semantic/context information than fastText, BERT, and Doc2Vec, which is visible through its improved performance across languages. It can be inferred that the LASER system for MaNLI gives good results that are equally comparable with XNLI results using LASER by Artetxe & Schwenk (2019) in Table 12.

**Table 12:** Test Accuracies of NLI for different languages using XNLI dataset.

Approach	en	fr	es	de	el	bg	ru	tr	ar	vi	th	zh	hi	sw	ur
BiLSTM	73.7	67.7	68.7	67.7	68.9	67.9	65.4	64.2	64.8	66.4	64.1	65.8	64.1	55.7	58.4
BERT	81.4		74.3	70.5					62.1			63.8			58.3
LASER	73.9	71.9	72.9	72.6	72.8	74.2	72.1	69.7	71.4	72.0	69.2	71.4	65.5	62.2	61.0

LASER uses a parallel corpora to train the encoder decoder model which thereby produces semantic representations of sentences irrespective of the language. The encoder model is language independent as no language information is provided. Thus it showed a higher performance for classification of sentence pairs in the Malayalam language.

## Discussion

From Tables 5–8, sentence representation using LASER sentence encoder encodes a better representation, which is depicted in the recall and precision values of the NLI system using LASER. This is mainly because using zero shot transfer from languages like English to low resource languages.

The higher performance of LASER can be justified by its joint training with languages both resource rich and resource poor resulting in better semantic representation. It is based on neural machine translation with the same encoder decoder model for training all the languages. From the encoder model only the sentence information is passed to the decoder model. Using byte pair encoding, LASER method obtains new representations for every word which is then utilized by the BiLSTM layers. Multilingual BERT is trained on Wikipedia text and uses mainly an aggregation of token, segment and position embeddings and obtains sentence embeddings from masked language model based pretraining with the [CLS] token as the sentence embeddings. The training using neural machine translation makes LASER performance much better than mBERT that uses masked language model and next sentence prediction as the pretraining tasks.

Though binary systems identify entailments and contradictory pairs, the system performance is reduced when the neutral class was added. The syntactic and semantic difference between entailment and neutral pairs is negligible, and systems were not able to identify that difference. For the inference problem, this small semantic difference between hypothesis is important to distinguish entailments and neutral pairs. From the results, LASER embeddings can distinguish between entailment pairs and neutral pairs in the dataset more effectively as this representation does not lose much information while encoding. LASER pretrained sentence encoders are much faster than BERT and fastText based systems and provide higher performance than other sentence encoders with lesser training time.

**Figure 6:**
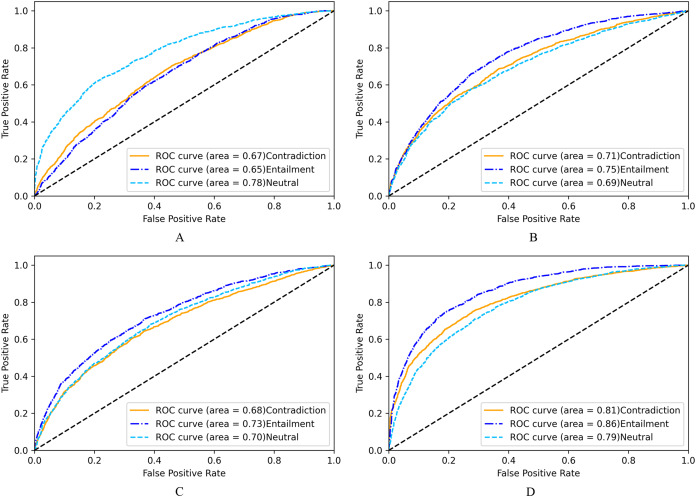
ROC for NLI multiclass classification. (A) Based on Doc2Vec. (B) Based on fastText. (C) Based on BERT. (D) Based on LASER.

From the receiver operating characteristics(ROC) curve in Fig. 6, the LASER based multiclass classification system (6D) has better classification in terms of AUC score of 0.81 for Contradiction class, 0.86 for Entailment class and 0.79 for Neutral class. Classifier using fastText (6B) obtained AUC scores of 0.71,0.75 and 0.69 for Contradiction, Entailment, and Neutral classes, respectively. BERT based system also has similar AUC values (6C). Doc2Vec performed poorly with lesser AUC scores(6A).

**Figure 7:**
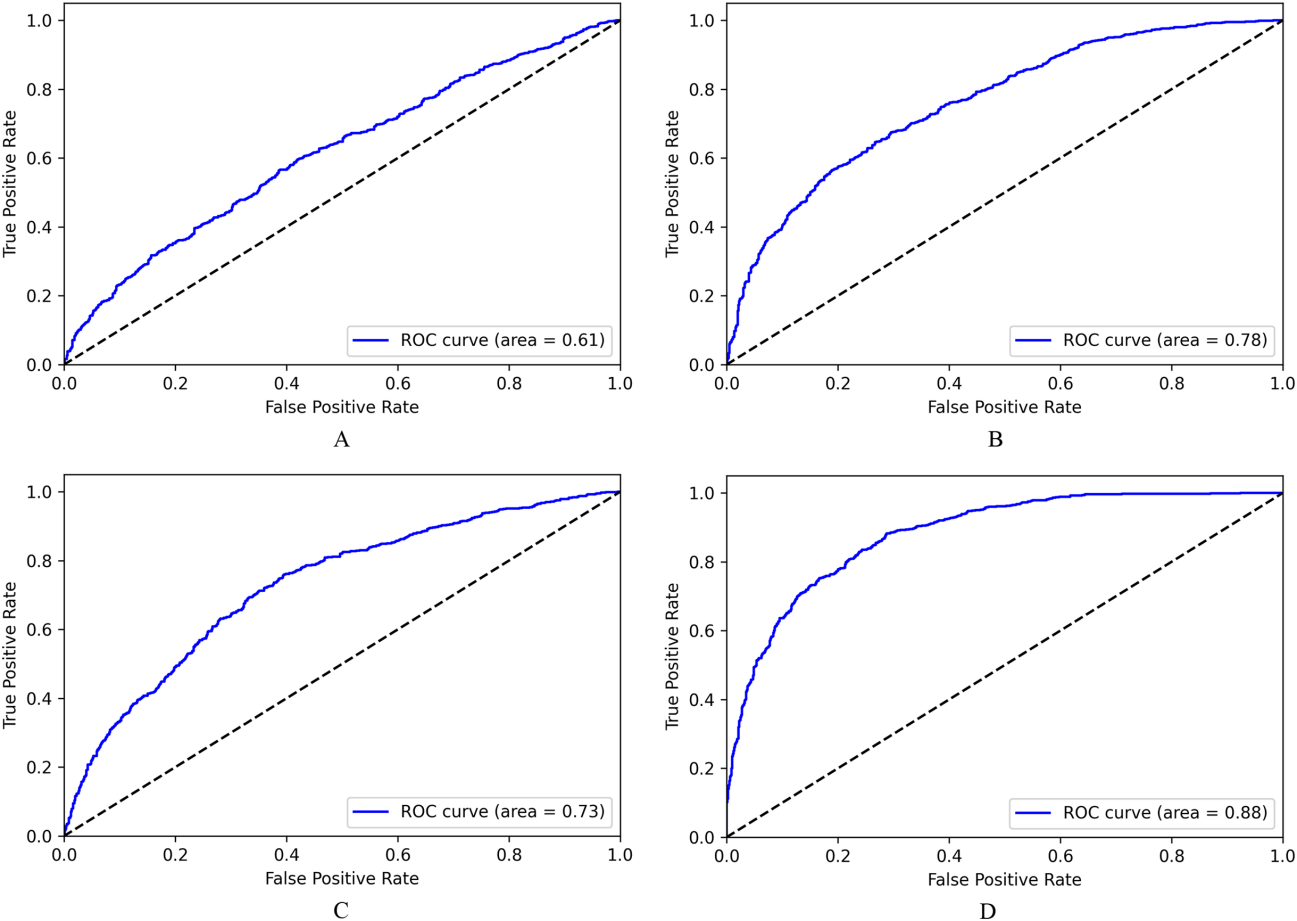
ROC for NLI binary classification. (A) Based on Doc2Vec. (B) Based on fastText. (C) Based on BERT. (D) Based on LASER.

We observe similar performance for binary classification in Fig. 7, where the LASER-based system (7D) has an AUC score of 0.88, and fastText based system (7B) has an AUC score of 0.78. Hence NLI classifier using LASER embeddings that substantially represent semantic information is better and can be used to embed sentences of any low resource languages, including Malayalam, without any fine-tuning of the encoder model.

**Figure 8:**
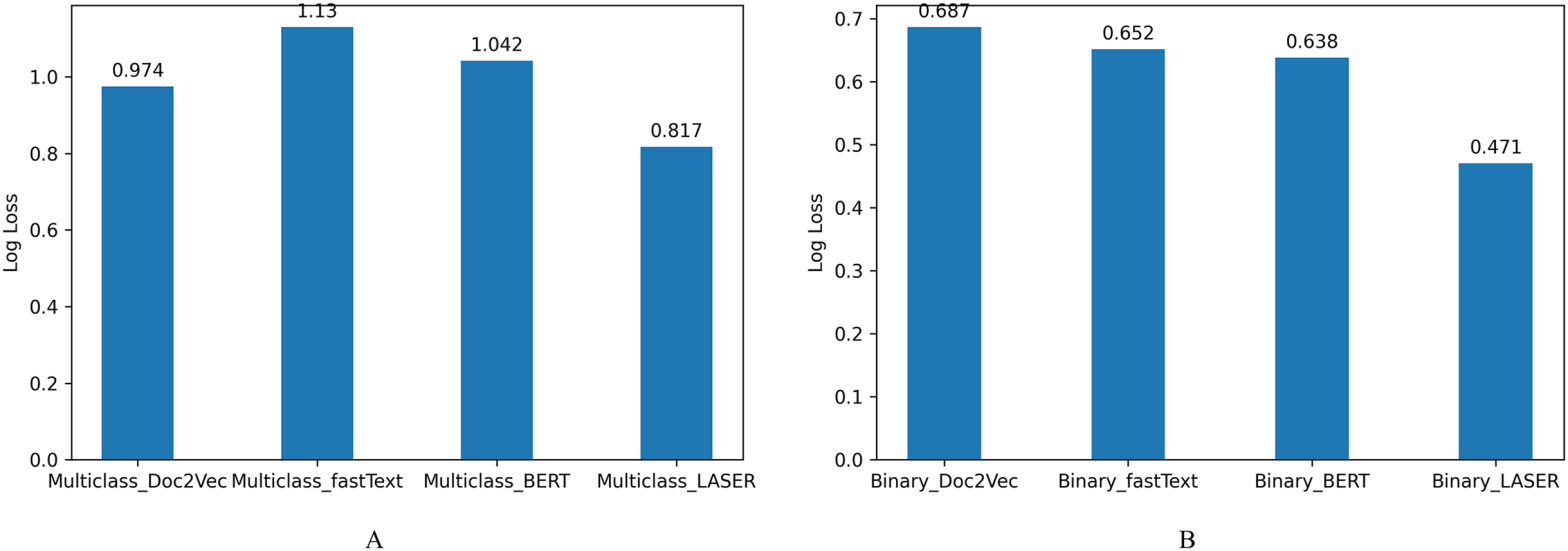
Log loss for NLI classification. (A) Multiclass NLI system. (B) Binary NLI system.

The log loss values represent the model’s unpredictability; Fig. 8 shows the loss values, indicating that the system’s predictability is also improved using efficient sentence representations. From these results, we understand that efficient sentence representations would help improve the predictability of the systems without increasing the complexity of the classifier models. LASER sentence encoders that embed premise and hypothesis reduced sentence encoding time and complexity of the classifier model compared with systems that encode sentences using fastText, BERT, and Doc2Vec.

### Error analysis

The error analysis includes quantitative analysis using confusion matrix and qualitative analysis of few incorrectly classified instances.

#### Quantitative analysis

We identified the number of correctly and incorrectly classified instances using Confusion matrix (https://scikit-learn.org/stable/modules/generated/sklearn.metrics.confusion_matrix.html), also called the error matrix. It is a table layout showing the performance of the model based on actual and predicted classes. The confusion matrix that shows the model performance and limitation is shown in Table 13 for multiclass classification. The binary classification resulted in confusion matrix shown in Table 14. This clearly shows high recall of LASER compared to other approaches for the Malayalam NLI task using the MaNLI dataset.

**Table 13:** Confusion matrix for multiclass classification.

(a) Doc2Vec based				
		Predicted		
		Contradict	Entail	Neutral
Actual	Contradict	329	902	420
	Entail	190	1,103	389
	Neutral	64	519	1,084

(b) FastText based				
		Predicted		
		Contradict	Entail	Neutral
Actual	Contradict	761	350	540
	Entail	300	898	484
	Neutral	295	318	1,054

(c) BERT based				
		Predicted		
		Contradict	Entail	Neutral
Actual	Contradict	858	666	127
	Entail	335	1,226	121
	Neutral	565	690	412

(d) LASER based				
		Predicted		
		Contradict	Entail	Neutral
Actual	Contradict	1,009	363	279
	Entail	143	1,313	226
	Neutral	392	379	896

**Table 14:** Confusion matrix for binary classification.

(a) Doc2Vec based			
		Predicted	
		Contradict	Entail
Actual	Contradict	352	446
	Entail	223	577

(b) FastText based			
		Predicted	
		Contradict	Entail
Actual	Contradict	395	403
	Entail	120	680

(c) BERT based			
		Predicted	
		Contradict	Entail
Actual	Contradict	521	277
	Entail	252	548

(d) LASER based			
		Predicted	
		Contradict	Entail
Actual	Contradict	517	281
	Entail	74	726

#### Qualitative analysis

**Figure 9:**
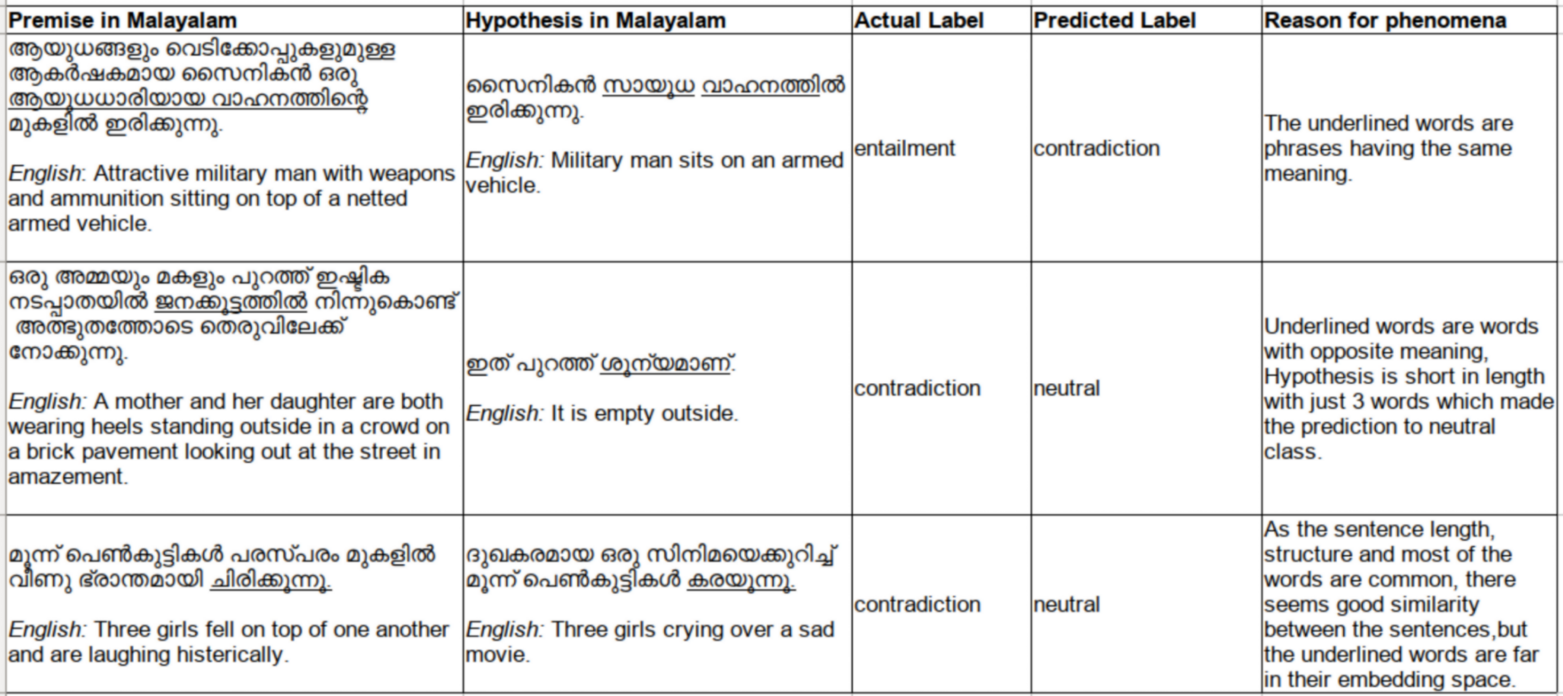
Sample incorrectly classified instances.

The samples of misclassified instances are given below in Fig. 9 which shows the challenging nature of this model with respect to the Malayalam language, having high agglutinating and inflectional properties. Phrasal verbs, implied meanings, similar sentences with single opposite word, the absence of background knowledge to derive implied meanings are few reasons for the incorrect classifications identified.

## Conclusion

Natural language inference is a task in language processing that has been addressed for many years. However, the primary focus was on languages like English, Chinese, and Japanese. Nevertheless, now there are attempts for NLI in other languages and even low resource languages. This paper’s proposed work is one such work that is the first attempt in the Malayalam language to the best of our knowledge. It is also an effort to understand the effectiveness of different sentence embeddings for NLI in Malayalam.

We also developed a translated dataset for NLI in Malayalam, without which we could not attempt this work. The dataset’s size is not a limiting factor nowadays, as there are pretrained models to use, thus enabling transfer learning in language systems with fewer resources. No external resources or knowledge bases like WordNet is used for inference identification, thus having a general approach applicable to other languages. The datasets for other low resource languages can also be developed using this approach.

In this work, we also compared different approaches Doc2Vec, FastText, mBERT and LASER, to conclude that LASER embeddings improved the system performance noticeably, emphasizing that improved input representations without much loss in information helped in obtaining better results. The system performance is compared with XNLI results. The encoder models publicly available are utilized in obtaining sentence representations. Being a language-agnostic model, it can be extended to other languages also.

## Supplemental Information

10.7717/peerj-cs.508/supp-1 Supplemental Information 1 Code for implementation.

## Additional Information and Declarations

### Data Availability

The following information was supplied regarding data availability: Data are available at GitHub: https://github.com/SaraRenG/MaNLI-data. Code for implementation is available in the Supplemental Files.
